# Supplementary material for: Resolving the full spectrum of human genome variation using Linked-Reads
Source: Genome Res. 2019 Apr;29(4):635–45. doi: 10.1101/gr.234443.118 (PMC6442396; doi:10.1101/gr.234443.118)
Supplement: Supplemental Material [file supp_gr.234443.118_Supplemental_longranger-2.2.zip › longranger-2.2.tar.bz2!/lr2.2-public/lib/stan/cmdstan/stan/doc/api/html/rev_2functions_2fmin_8hpp_source.html]

Stan: src/stan/agrad/rev/functions/fmin.hpp Source File


|  |  |
| --- | --- |
|  | Stan  2.5.0  probability, sampling & optimization |


- Main Page
- Related Pages
- Namespaces
- Classes
- Files

- File List
- File Members

All Classes Namespaces Files Functions Variables Typedefs Enumerations Enumerator Friends Macros Pages

- src
- stan
- agrad
- rev
- functions

fmin.hpp

Go to the documentation of this file.

1 #ifndef STAN\_\_AGRAD\_\_REV\_\_FUNCTIONS\_\_FMIN\_HPP

2 #define STAN\_\_AGRAD\_\_REV\_\_FUNCTIONS\_\_FMIN\_HPP

3

4 #include <stan/agrad/rev/var.hpp>

5 #include <boost/math/special\_functions/fpclassify.hpp>

6 #include <stan/agrad/rev/internal/precomp\_v\_vari.hpp>

7 #include <stan/agrad/rev/internal/precomputed\_gradients.hpp>

8 #include <stan/math/constants.hpp>

9 #include <stan/meta/likely.hpp>

10

11 namespace stan {

12  namespace agrad {

13

60  inline var fmin(const stan::agrad::var& a,

61  const stan::agrad::var& b) {

62  if (unlikely(boost::math::isnan(a.vi\_->val\_))) {

63  if(boost::math::isnan(b.vi\_->val\_)) {

64  std::vector<stan::agrad::var> vars;

65  std::vector<double> grads;

66  vars.push\_back(a);

67  vars.push\_back(b);

68  grads.push\_back(stan::math::NOT\_A\_NUMBER);

69  grads.push\_back(stan::math::NOT\_A\_NUMBER);

70  return var(precomputed\_gradients(stan::math::NOT\_A\_NUMBER,

71  vars, grads));

72  }

73  else

74  return b;

75  } else if (unlikely(boost::math::isnan(b.vi\_->val\_)))

76  return a;

77  else

78  return a.vi\_->val\_ < b.vi\_->val\_ ? a : b;

79  }

80

95  inline var fmin(const stan::agrad::var& a,

96  const double& b) {

97  if (unlikely(boost::math::isnan(a.vi\_->val\_))) {

98  if(boost::math::isnan(b))

99  return var(new precomp\_v\_vari(stan::math::NOT\_A\_NUMBER,

100  a.vi\_,

101  stan::math::NOT\_A\_NUMBER));

102  else

103  return var(b);

104  } else if (unlikely(boost::math::isnan(b)))

105  return a;

106  else

107  return a.vi\_->val\_ <= b ? a : var(b);

108  }

109

126  inline var fmin(const double& a,

127  const stan::agrad::var& b) {

128  if (unlikely(boost::math::isnan(b.vi\_->val\_))) {

129  if(boost::math::isnan(a))

130  return var(new precomp\_v\_vari(stan::math::NOT\_A\_NUMBER,

131  b.vi\_,

132  stan::math::NOT\_A\_NUMBER));

133  else

134  return var(a);

135  } else if (unlikely(boost::math::isnan(a)))

136  return b;

137  else

138  return a < b.vi\_->val\_ ? var(a) : b;

139  }

140

141  }

142 }

143 #endif

boost::math::isnan

bool isnan(const stan::agrad::var &v)

Checks if the given number is NaN.

**Definition:** boost\_fpclassify.hpp:69

precomp\_v\_vari.hpp

stan::math::NOT\_A\_NUMBER

const double NOT\_A\_NUMBER

(Quiet) not-a-number value.

**Definition:** constants.hpp:53

stan::agrad::precomputed\_gradients

var precomputed\_gradients(const double value, const std::vector< var > &vars, const std::vector< double > &gradients)

This function is provided for Stan users that want to compute gradients without using Stan's auto-dif...

**Definition:** precomputed\_gradients.hpp:86

stan::agrad::vari::val\_

const double val\_

The value of this variable.

**Definition:** vari.hpp:37

stan::agrad::var::vi\_

vari \* vi\_

Pointer to the implementation of this variable.

**Definition:** var.hpp:40

unlikely

#define unlikely(x)

**Definition:** likely.hpp:9

stan::agrad::fmin

fvar< T > fmin(const fvar< T > &x1, const fvar< T > &x2)

**Definition:** fmin.hpp:17

var.hpp

likely.hpp

precomputed\_gradients.hpp

stan::agrad::precomp\_v\_vari

**Definition:** precomp\_v\_vari.hpp:11

stan::agrad::var

Independent (input) and dependent (output) variables for gradients.

**Definition:** var.hpp:27

constants.hpp


---

[ Stan Home Page ]

*© 2011–2014,
Stan Development Team.*
